# Design and implementation of a prototype head and neck phantom for the performance evaluation of gamma imaging systems

**DOI:** 10.1186/s40658-017-0186-3

**Published:** 2017-07-06

**Authors:** Mohammed S. Alqahtani, John E. Lees, Sarah L. Bugby, Piyal Samara-Ratna, Aik H. Ng, Alan C. Perkins

**Affiliations:** 1 Space Research Centre, Department of Physics and Astronomy, University of Leicester, Leicester, LE1 7RH UK; 2 Radiological Sciences Department, College of Applied Medical Sciences, King Khalid University, Zip code 61481, Abha, 3665 Kingdom of Saudi Arabia; 3 Radiological Sciences, School of Medicine, University of Nottingham, Nottingham, NG7 2UH UK; 4 Medical Physics and Clinical Engineering, Nottingham University Hospitals NHS Trust, Nottingham, UK

**Keywords:** Sentinel lymph nodes detection, SFOV gamma camera, Head and neck phantom, Thyroid phantom, 3D printing, Thyroid scan, Anthropomorphic phantom, SPECT

## Abstract

**Background:**

A prototype anthropomorphic head and neck phantom has been designed to simulate the adult head and neck anatomy including some internal organs and tissues of interest, such as thyroid gland and sentinel lymph nodes (SLNs). The design of the head and neck phantom includes an inner jig holding the simulated SLNs and thyroid gland. The thyroid gland structure was manufactured using three-dimensional (3D) printing taking into consideration the morphology and shape of a healthy adult thyroid gland.

**Result:**

The head and neck phantom was employed to simulate a situation where there are four SLNs distributed at two different vertical levels and at two depths within the neck. Contrast to noise ratio (CNR) calculations were performed for the detected SLNs at an 80 mm distance between both pinhole collimators (0.5 and 1.0 mm diameters) and the surface of the head and neck phantom with a 100 s acquisition time. The recorded CNR values for the simulated SLNs are higher when the hybrid gamma camera (HGC) was fitted with the 1.0 mm diameter pinhole collimator. For instance, the recorded CNR values for the superficially simulated SLN (15 mm depth) containing 0.1 MBq of ^99m^Tc using 0.5 and 1.0 mm diameter pinhole collimators are 6.48 and 16.42, respectively (~87% difference).

Gamma and hybrid optical images were acquired using the HGC for the simulated thyroid gland. The count profiles through the middle of the simulated thyroid gland images provided by both pinhole collimators were obtained. The HGC could clearly differentiate the individual peaks of both thyroid lobes in the gamma image produced by the 0.5-mm pinhole collimator. In contrast, the recorded count profile for the acquired image using the 1.0-mm-diameter pinhole collimator showed broader peaks for both lobes, reflecting the degradation of the spatial resolution with increasing the diameter of the pinhole collimator.

**Conclusions:**

This anthropomorphic head and neck phantom provides a valuable tool for assessing the imaging ability of gamma cameras used for imaging the head and neck region. The standardisation of test phantoms for SFOV gamma systems will provide an opportunity to collect data across various medical centres. The phantom described is cost effective, reproducible, flexible and anatomically representative.

## Background

Nuclear medicine procedures are widely used in the diagnosis and therapy of various pathologies of the head and neck. Traditionally, nuclear medicine has been employed in the diagnosis and therapy of both benign and malignant thyroid diseases. In addition, there has been much recent attention to preoperative lymphoscintigraphic imaging for melanoma, oral cancer and parotid gland carcinomas is a well-recognised procedure that has contributed to the improvement of surgical outcomes [1, 2]. Imaging is used to identify lymphatic drainage paths and locate sentinel lymph nodes (SLNs) that may contain disseminated disease. SLN mapping and biopsy in the head and neck usually follow preoperative lymphoscintigraphic imaging [3]. This procedure is used to determine the status of SLNs, which has been shown to provide crucial prognostic details of metastatic growth [4].

Performing preoperative lymphoscintigraphic imaging and SLN mapping procedures in the head and neck region is challenging in terms of anatomy [5]. The head and neck region contains several hundred potential lymph nodes that are distributed over different depths and have numerous different lymphatic suppliers, which complicates tracing the lymphatic drainage mechanisms [6]. Furthermore, the natural anatomical and physiological intricacies of the lymphatic network in the head and neck region pose technical difficulties in the case of preoperative lymphoscintigraphy and SLN mapping procedures. As structures in the head and neck are so closely packed, it is common for the large amount of radioactivity in the injection site to mask the signal from nearby SLNs [7]. Head and neck anatomy is complex and variable, with lymphatic drainage pattern differing between patients; 4% of SLNs detected related to oral cavity tumours are on the contralateral of the neck [8]. The identification of sentinel lymph nodes can also be hampered by the rapid displacement of the intradermally injected radioisotope, which may accumulate in several lymph nodes within a short period.

The use of an additional visual guided system can enhance the interventional process in SLN surgery pertaining to the head and neck region. This is seen in the case of small field of view (SFOV) portable gamma cameras used during radio-guided surgery. These provide visual guidance during critical SLN biopsy processes and can help surgeons in acquiring real-time, intraoperative lymphoscintigraphic images [9]. The intraoperative usefulness of these cameras has been indicated in several scientific studies [10–13], including studies of head and neck SLN mapping. However, there remains a need for quality control procedures for testing the use of these portable gamma imaging systems in specific clinical procedures.

Quality assurance and performance testing of any medical imaging device commonly involves the use of phantoms, which are structured to imitate the process of radiation interaction with human tissues [14]. Medical phantoms are designed and developed in different forms ranging from simple cylindrical or cubic shaped objects to the accurate three-dimensional (3D) representation of body and organ shape. Tissue-equivalent anthropomorphic phantoms that present the true shape of human body parts are useful in various disciplines of radiology and radiation dosimetry, including therapeutic and diagnostic procedures [15–17].

This study aims to develop and standardise a test object and protocols particularly suited for small field of view (SFOV) compact gamma cameras but which can be equally useful for large field of view (LFOV) systems. A flexible insert was designed for a commercially available head and neck phantom. This insert effectively imitates human tissues in that region including simulating SLNs and a life-size thyroid gland. The phantom insert was designed to be reproducible at relatively low costs; allowing this test object to be incorporated into different quality assurance protocols. It is proposed that this phantom can be employed in assessing the clinical usefulness of SFOV gamma imaging systems in SLN mapping and small organ imaging.

## Methods

### Head and neck phantom

**Fig. 1:**
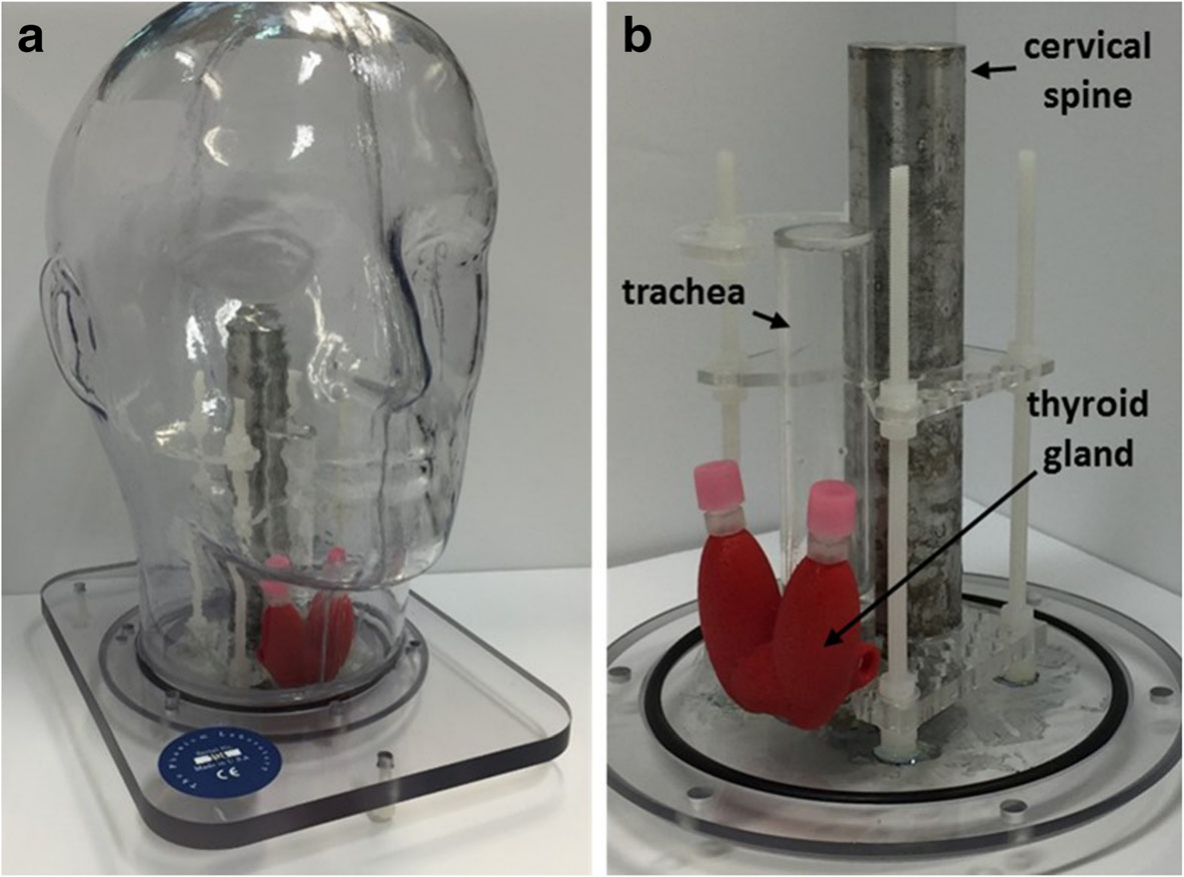
**a** Photograph of the head and neck phantom. **b** The internal jig with the attached thyroid phantom from anterolateral view **b**

The outer shell of the phantom was obtained from The Phantom Laboratory [18]. This comprised a shell manufactured from ~3.2-mm-thick cellulous acetate butyrate (CAB) mounted on a polycarbonate end plate, a transparent plastic selected because of its low water absorption and its strength. The anthropomorphic head and neck phantom is constructed to allow filling with water or other tissue equivalent liquids. The shape of the outer shell imitates the head and neck contours of an average size adult male; the maximum axial height being 255 mm, and the maximum transverse width 175 mm (Fig. 1a).

**Fig. 2:**
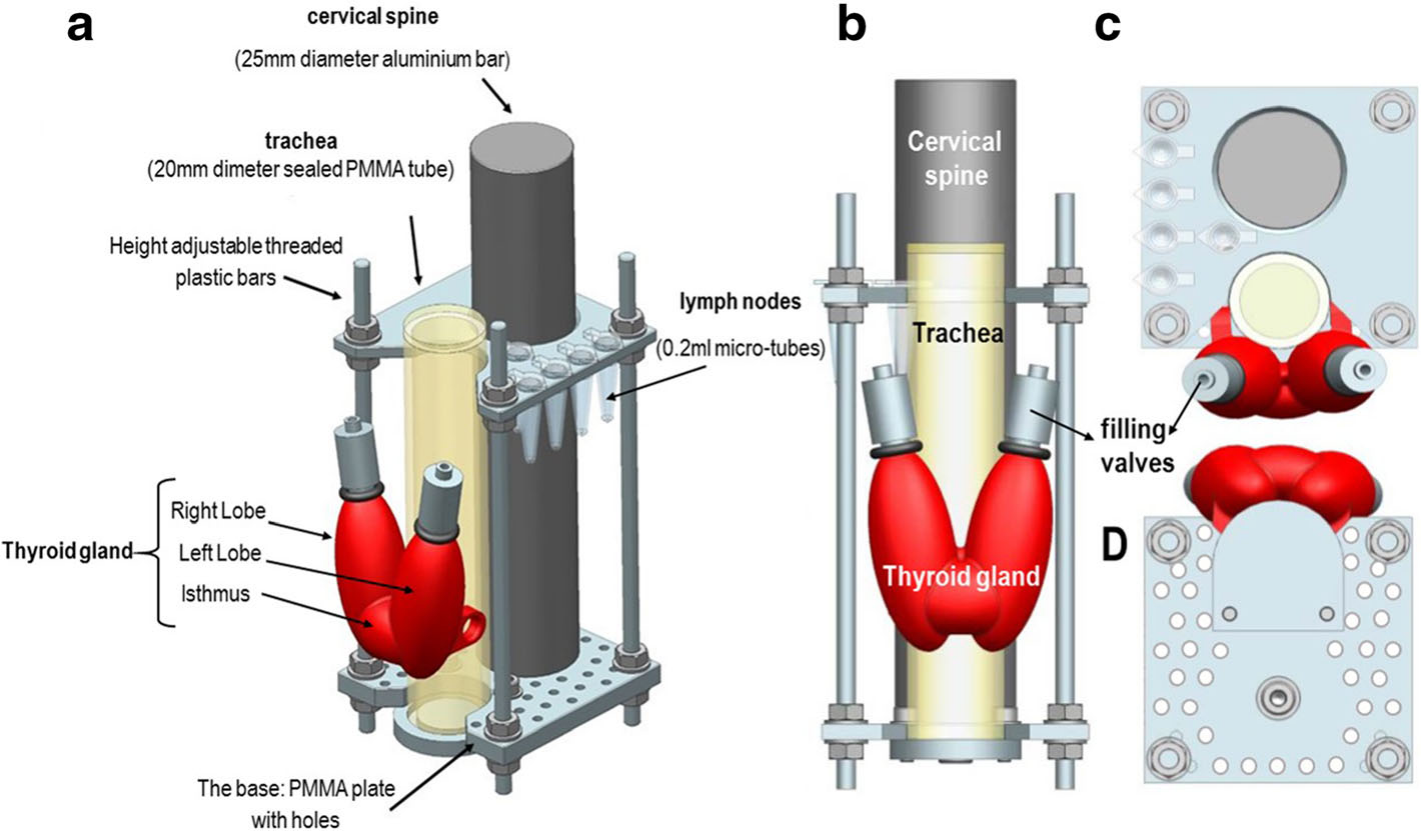
Schematics of the internal jig showing the mounted simulated thyroid gland (*red*) and simulated sentinel lymph nodes (SLNs). The shape and position of the thyroid phantom relative to the simulated trachea: anterolateral (**a**), anterior (**b**), superior (**c**), and inferior (**d**) views

An anatomical insert (further details discussed below, see Figs. 1 and 2) was used in conjunction with the outer shell of the phantom. This insert was modular and could include a simulated thyroid gland, trachea, cervical spine, lymph nodes and injection sites in any combination together with various locations for lymph nodes and injection sites.

#### Anatomical simulation

Information about the anatomical characteristics of the human neck has been collected and analysed, and tissue equivalent materials were selected for mimicking soft, cartilaginous and bone tissues. The size of a thyroid of a healthy adult was chosen for simulation. Various studies have been published summarising the geometric information of the healthy thyroid from medical imaging investigations [19–25]. The average geometric measurement of healthy male thyroid glands given in these papers was considered to design the thyroid phantom (Table 1).

**Table 1:** Summary of the anthropomorphic thyroid phantom parameters

Outer shell thickness (mm)	Internal lobe dimensions (mm)			Internal isthmus dimensions (mm)		
	Vertical length	Maximum width	Maximum depth	Vertical length	Maximum width	Maximum depth
3	42	17.5	10	12	15	5

Once the size had been determined, the structure was manufactured using 3D printing facility (Fig. 2). A red coloured acrylonitrile butadiene styrene (ABS) thermoplastic polymer material (chemical formula: (C_8_H_8_ · C_4_H_6_ · C_3_H_3_N)_n_) was used to manufacture the outer shell of the thyroid insert; the inner sealed space was filled with water mixed with the desired radioactivity concentration. Two valves allowed filling the inner space with the radioactive solution via a syringe (Fig. 2).

A simulated trachea was constructed to hold the life-size thyroid phantom. The trachea was designed and manufactured as a sealed cylinder filled with air and was made of polymethyl methacrylate (PMMA), chemical formula: (C_5_O_2_H_8_)_n,_ (height = 150 mm, diameter = 20 mm, thickness = 3 mm). The anthropomorphic thyroid phantom was manually attached to the trachea simulator in its appropriate anatomical position. The internal jig also included a cylindrical rod of aluminium that was used to imitate the cervical spine in the neck region (height = 200 mm, diameter = 25 mm). The materials chosen to simulate the head and neck parts of the phantom show an acceptable degree of similarity to normal human tissues as characterised in a number of published studies (see Table 2).

**Table 2:** Comparison of the densities for the materials utilised for the phantom designing with real human tissues [41–47]

Materials	Density (g/cm^3^)	Mass attenuation coefficient (cm^2^/g)	Calculated Hounsfield unit (HU)
Water	1	0.154	0
Thyroid gland	0.98	–	–
ABS thermoplastic polymer	1.06–1.08	0.152	46–66
Bone tissues	1.7–2.0	0.156	722–1026
Aluminium	2.69	0.137	1393
Soft tissues	1.04	0.153	33
PMMA	1.18	0.149	142
Trachea	0.98–1.1	–	–

#### Sentinel lymph nodes (SLN) and injection site simulation

Low profile plastic micro-tubes (0.2 ml) with attached caps have been used to simulate SLNs or injection sites. These are easily removed from the phantom (along with the thyroid) for easy storage while any activity decays. These tubes can be filled with various activity concentrations to simulate a range of clinical scenarios, e.g. high activity for an injection site and low activity for a SLN.

**Fig. 3:**
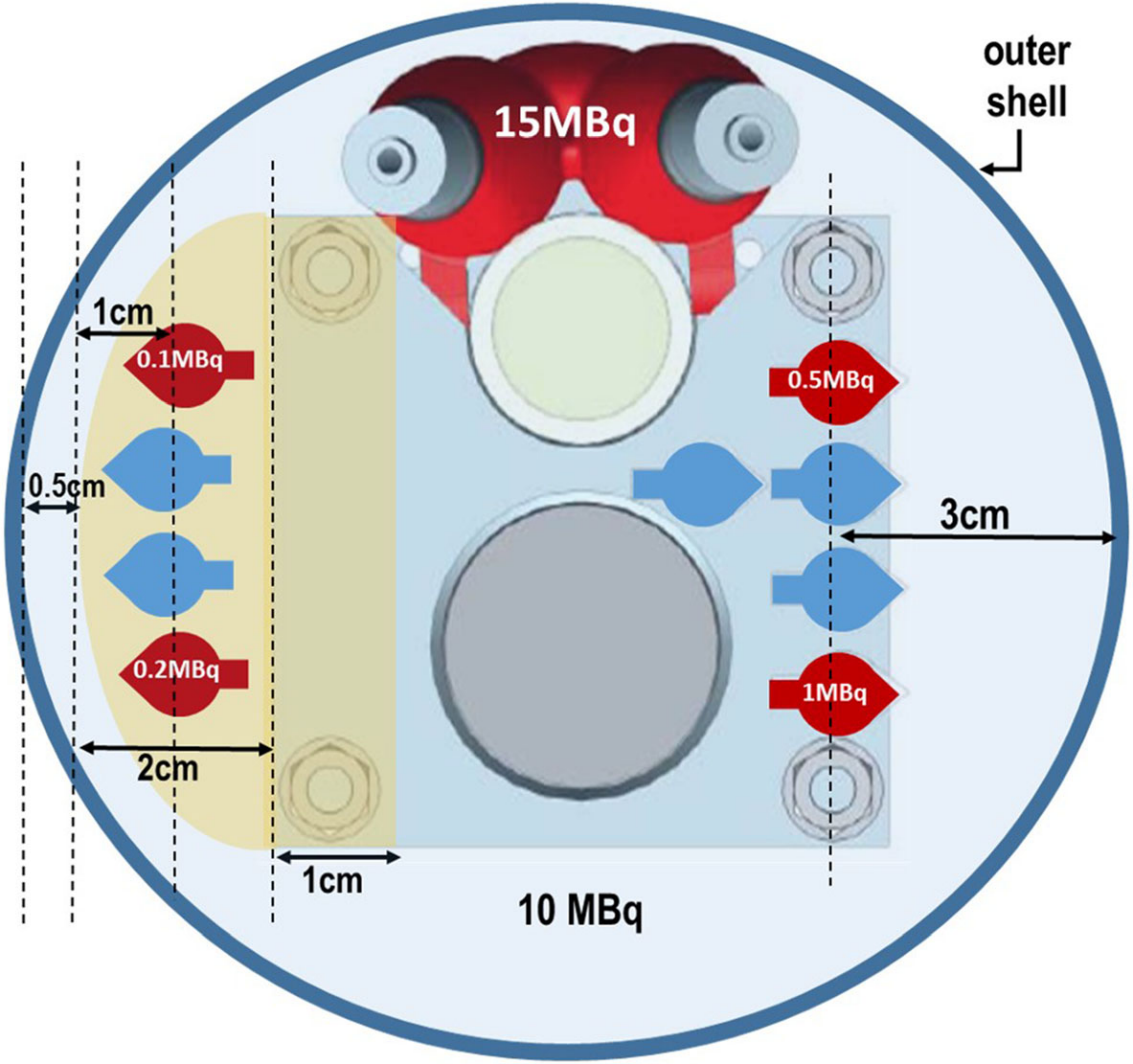
A schematic diagram showing a cross section through the neck region of the phantom; it shows the position, lateral depth and amount of radioactivity in the simulated lymph nodes

A selection of PMMA plates, 3 mm thick, were designed, containing drilled holes to hold the micro-tubes, which attach directly to the insert (Fig. 2). Node placement can be varied by adjusting the height of the plates on the insert, varying the shape of plate used (see Fig. 3 for sample shapes) and by changing the hole a node is set in. For instance, deep, posterior and superficial cervical lymph nodes can be simulated and located in their accurate anatomical place and can be individually filled with different radioactivity concentrations. The phantom design enables reproducible node placements for different applications.

### Radioactivity distribution and imaging procedure

When simulating a scintigraphic procedure, the amounts of radioactivity should be a true reflection of the activity concentrations reached in clinical situations. In practice, identifying the particular radioactive material concentration for SLNs in the head and neck region can be a difficult task due to the different nature of radiopharmaceuticals and patient anatomy and physiology.

For this study, 4 simulated SLNs were placed at superficial (15 mm depth) and deep (30 mm depth) locations in the head and neck region with various concentrations of ^99m^Tc solution ranging between 0.1 and 1 MBq, as shown in Fig. 3. These concentrations have been selected with the guidance of available clinical data [26–31]. The uptake of ^99m^Tc pertechnetate in the thyroid gland is considered to be between 1 to 5% of administrated activities ranging between 185 and 370 MBq [32]; from these figures, 15 MBq of ^99m^Tc was taken as an appropriate amount of activity to represent the radioactivity uptake in the thyroid gland.

The average amount of activity usually administrated for head and neck lymphoscintigraphy is 20 MBq [33], and it was assumed that half of the injected activity will be distributed in the tissues surrounding the targeted SLNs. This background activity was simulated through mixing 10 MBq of ^99m^Tc solution with the water filling the outer shell of the head and neck phantom.

### SPECT-CT imaging

The phantom was also investigated using a Philips BrightView XCT dual head SPECT-CT system in the nuclear medicine clinic at Queen’s Medical Centre, Nottingham [34]. The phantom was fastened to the patient table with the patient head support in place. The camera was fitted with a low energy parallel collimator, and image data was acquired in a 128 × 128 matrix through 120 angular increments each of 20 s per angle over a 360° rotation. The data were processed on a dedicated nuclear medicine computer (Hermes Medical Solutions, London, UK). Reconstruction was performed using the Ordered-Subsets Expectation Maximisation (OSEM) with 15 subsets and with 4 iterations and displayed in sagittal, transverse and coronal planes. The reconstructed images were filtered with a 3D Gaussian function having a full width at half maximum of 8 mm. CT imaging was performed with 120 kVp and 20 mAs.

### SFOV imaging

The Hybrid Gamma Camera (HGC), designed and manufactured at University of Leicester, UK, is a novel SFOV portable gamma imager. The gamma detector consisted of a caesium iodide doped with thallium (CsI(Tl)) scintillator (1500 μm thick) coupled to an electron multiplying charge-coupled device (EMCCD) (i.e. e2v CCD97 BI) and a tungsten pinhole collimator. The recorded intrinsic spatial resolution for the CsI(Tl) scintillator is 0.23 ± 0.02 mm, and it has a sensitivity of 40 ± 3% at 140.5 keV. Detailed information of the HGC design, manufacture and characteristics are available elsewhere [35–37]. Two pinhole collimators (0.5 and 1.0 mm diameter) were fitted to the camera interchangeably during the study. Various collimator-to-surface distances (ranging between 80 and 200 mm) were selected to produce results with a range of field of views.

**Fig. 4:**
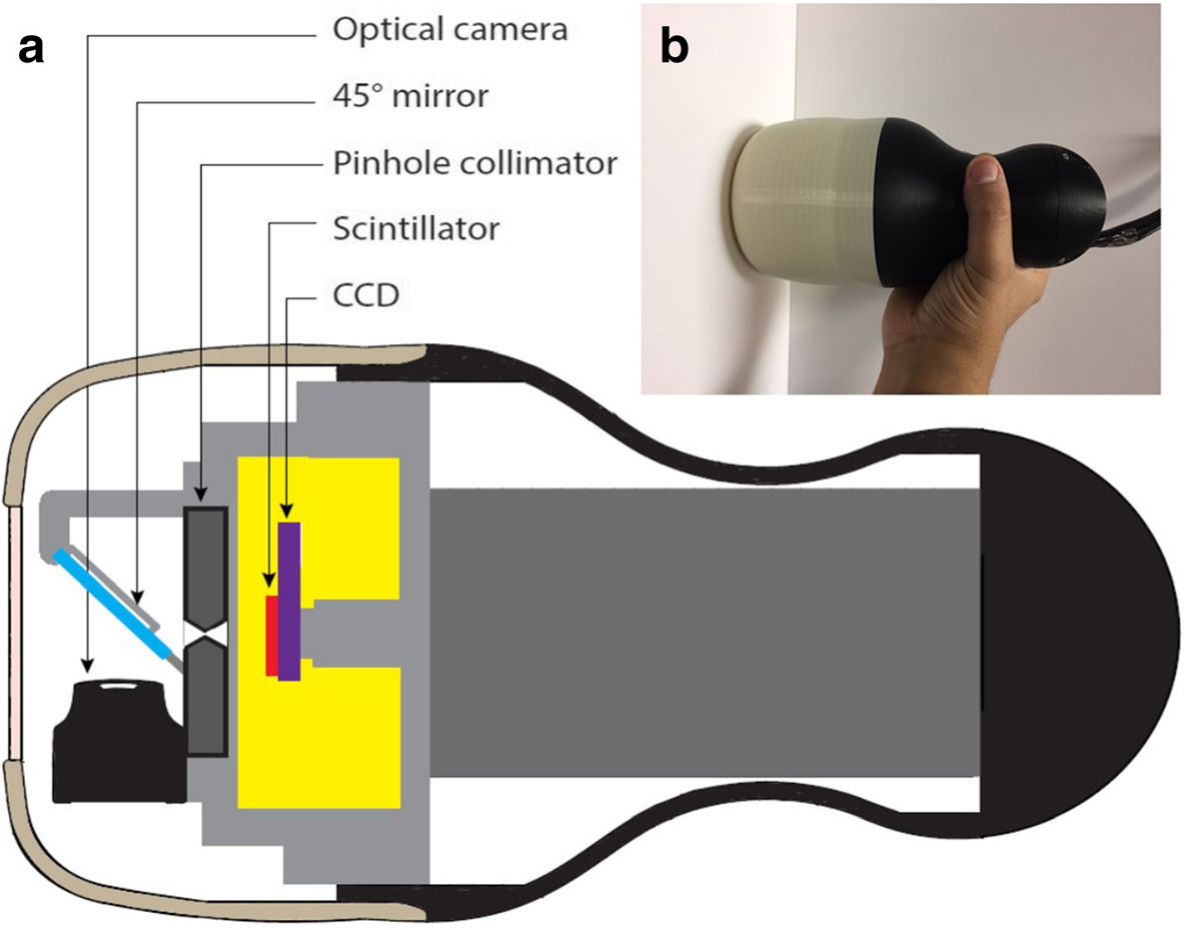
**a** A schematic diagram for the hybrid gamma camera (HGC) showing its internal structure. **b** Photograph of the HGC

The hybrid optical-gamma camera is designed in a configuration that allows simultaneous hybrid imaging for the targeted tissues with different imaging outputs (i.e. optical, gamma, or hybrid), as seen in Fig. 4. An additional hybrid optical-gamma anterior view of the neck region was acquired to illustrate the localisation information provided by fused images.

For the gamma images produced by the HGC, count profiles were acquired for the simulated thyroid gland gamma images at a collimator-to-surface distance of 120 mm using both pinhole collimators. Furthermore, two circular regions of interest (ROIs) were identified corresponding to the simulated SLN size to obtain the contrast and noise values. The contrast was then calculated as the difference between node and background ROI mean count values, with noise being defined as the standard deviation in the background ROI for calculating the contrast to noise ratio (CNR) of the simulated SLN, as explained in detail elsewhere [37]. The ability of the HGC to detect different targeted nodes is evaluated following Rose’s approximation [38]; a node within the FOV is classified as a detected node when its CNR value set above 3–5.

## Results

### SPECT and SPECT-CT imaging

**Fig. 5:**
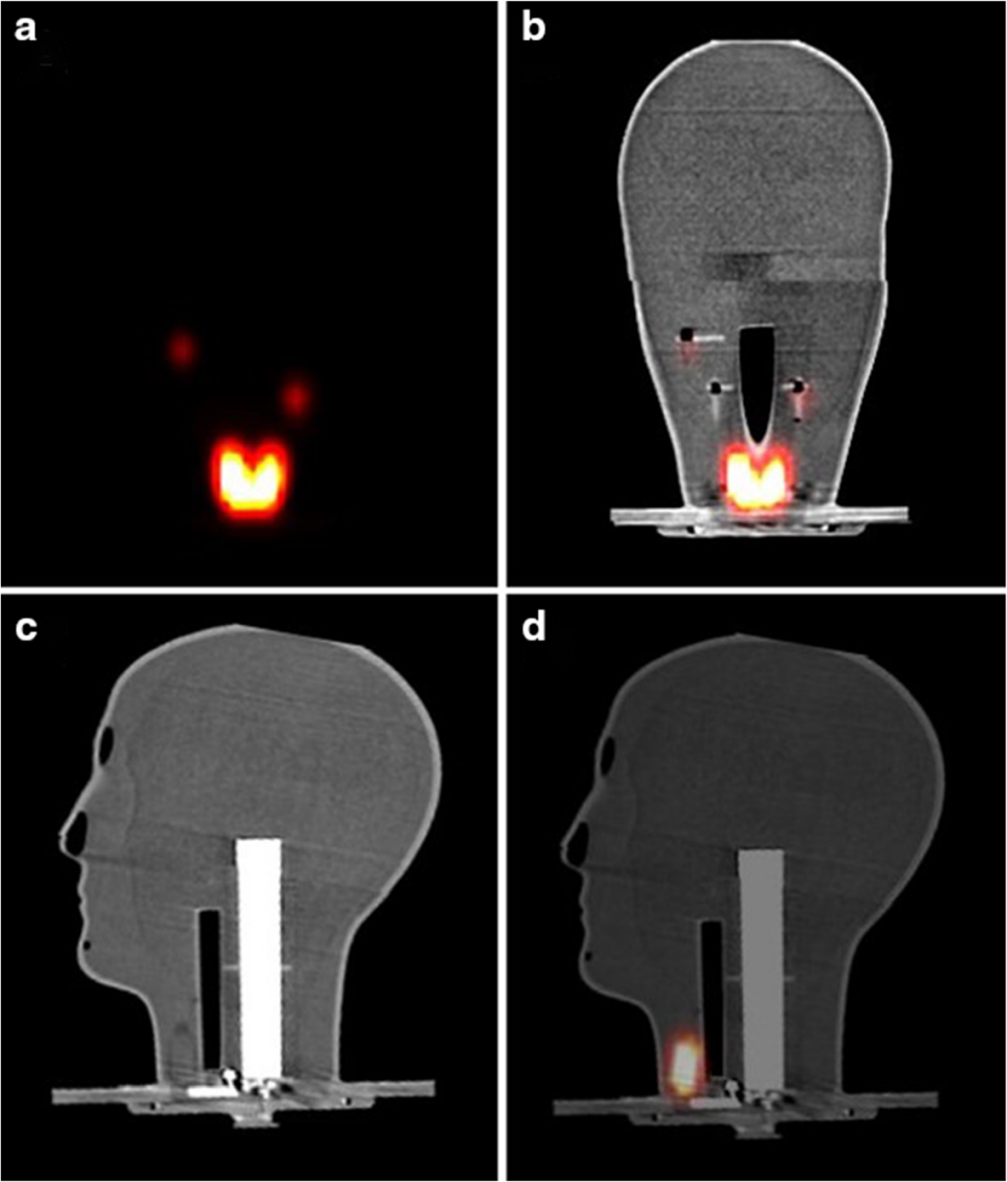
Coronal SPECT and SPECT-CT images showing the position of the simulated SLNs (0.1–0.5 MBq) and the simulated thyroid gland (15 MBq) within the phantom (**a**, **b**); CT and SPECT-CT images of the midsagittal plane of the head and neck phantom showing the simulated thyroid gland, trachea and cervical spine (**c**, **d**)

SPECT-CT images were used to validate the anatomical structure of the phantom. The appearances of the simulated thyroid gland in the coronal plane images were similar to those of a healthy thyroid (Fig. 5a, b). Furthermore, the accurate positioning of the simulated thyroid gland and the uniform distribution of radioactivity throughout both lobes and the isthmus reflect the design accuracy of the phantom and its suitability for various gamma imaging performance tests.

Figure 5c, d shows the SPECT-CT images through the midsagittal plane of the phantom. These images show the structure of the internal jig components including the simulated cervical spine, trachea and thyroid gland. A variation in X-ray contrast, as demonstrated in the images, for different simulated anatomical structures is in line with the proposed appearance of these structures in real life, which proves the suitability of the materials used to simulate the outer shell and the internal parts of the phantom.

**Fig. 6:**
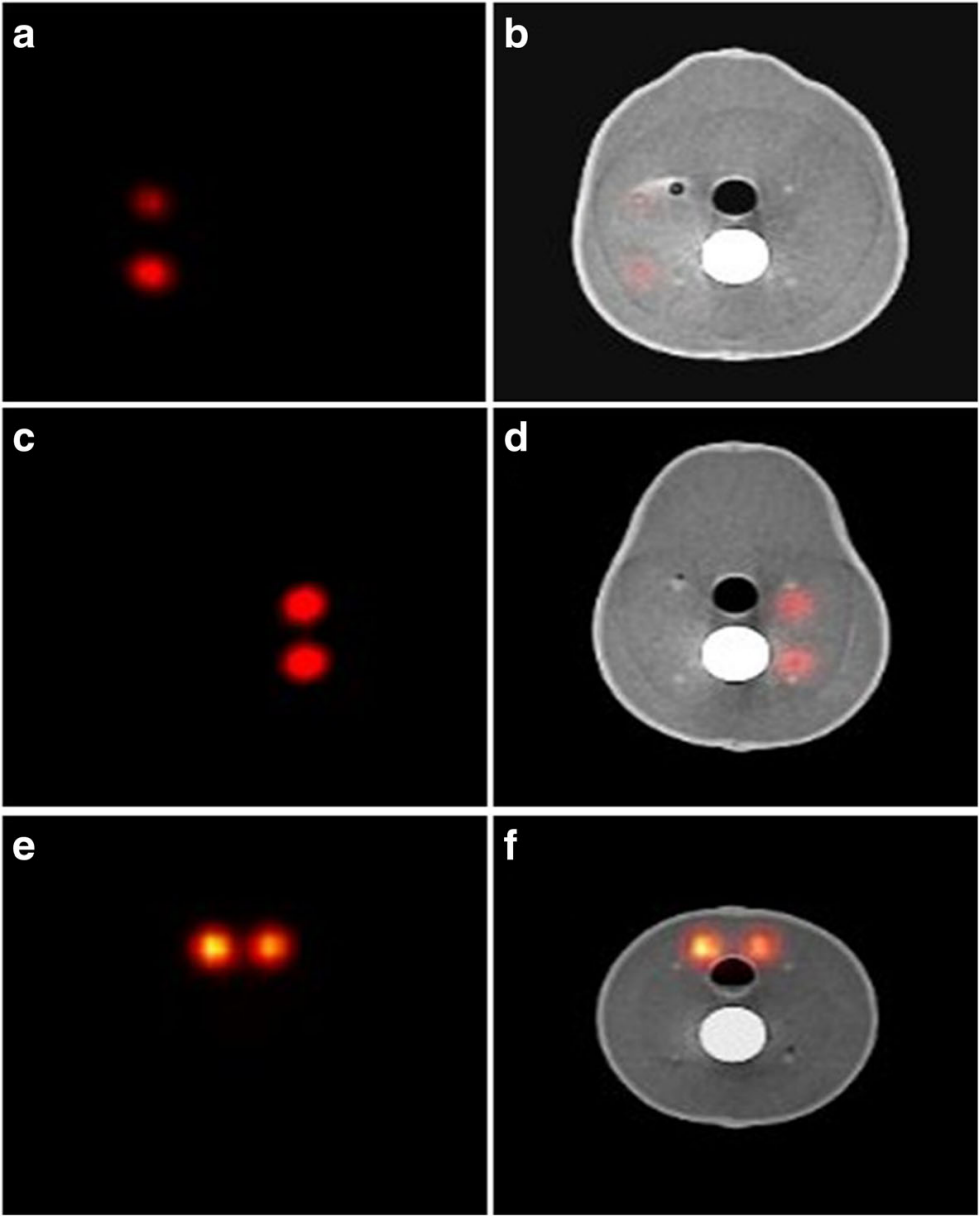
SPECT and SPECT-CT images in the transverse plane representing the anatomical structure of the head and neck phantom; images **a** and **b** show two superficially low activity uptake simulated SLNs in gamma and hybrid modes (0.1 and 0.2 MBq, respectively). Images **c** and **d** show two deeper suited, higher activity uptake simulated SLNs in gamma and hybrid modes (0.5 and 1.0 MBq, respectively). Images **e** and **f** show the thyroid level SPECT and SPECT-CT images in the neck region (15 MBq)

In Fig. 6, two simulated superficial SLNs were placed in the area of the parotid gland, having two different low activity concentrations, to simulate targeted parotid SLNs (Fig. 6 a, b). In Fig. 6c, d, deep simulated cervical SLNs (i.e. 30 mm depth) were located in their accurate anatomical position taking into account the position of the simulated trachea and cervical spine. Both lobes of the simulated thyroid gland can be identified in the proper position attached to the simulated trachea in a way that can provide valuable information about the capability of different gamma scanning systems in imaging the thyroid gland, Fig. 6e, f.

**Fig. 7:**
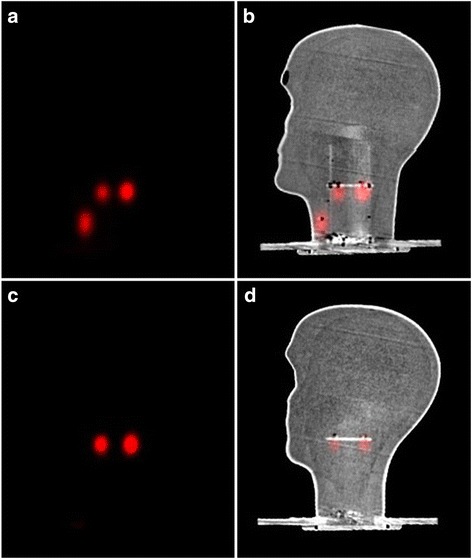
SPECT and SPECT-CT images in the sagittal plane showing the deeper simulated SLNs (0.5–1.0 MBq) and the simulated thyroid gland in **a** and **b**; images **c** and **d** show the simulated superficial SLNs at the parotid gland level (0.1–0.2 MBq)

The simulated SLNs were also imaged through the sagittal plane. SPECT and SPECT-CT images, in the sagittal plane, were acquired to represent the deep SLNs (30 mm depth) in the cervical region (Fig. 7a, b) and the superficial SLNs (i.e. 15 mm depth) in the parotid region (Fig. 7c, d).

### SFOV hybrid gamma camera (HGC) imaging

The anthropomorphic head and neck phantom was employed to show the capabilities of the HGC for imaging small organs and mapping SLNs in the head and neck region. The imaging protocol utilised, as well as the anthropomorphic phantom, is suitable for different SFOV gamma imaging systems and the experimental setup can be replicated for comparison purposes.

#### Thyroid phantom images

**Fig. 8:**
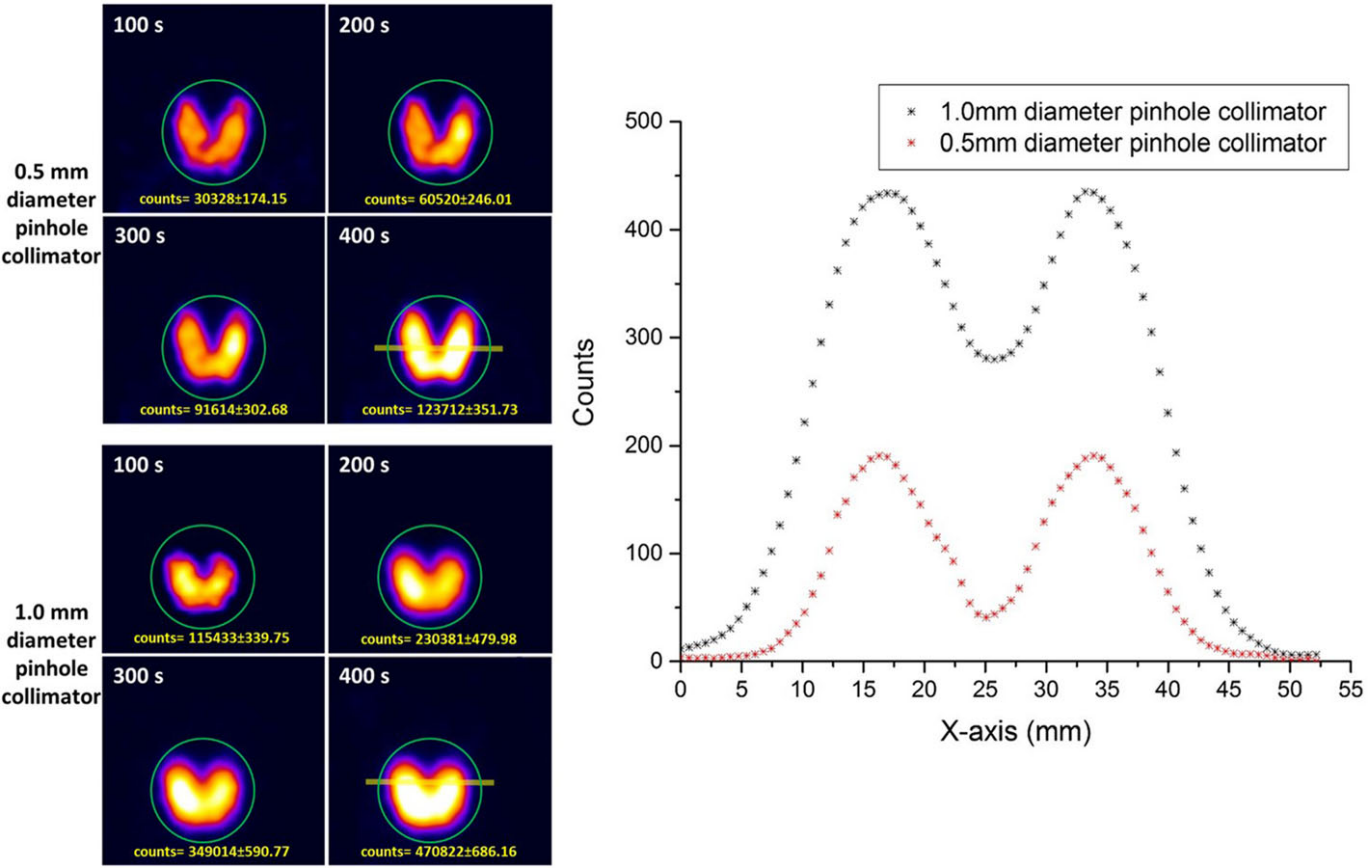
*Left-hand side*: planar HGC gamma images of the simulated thyroid gland at a 120 mm collimator-to-surface distance acquired with different acquisition times ranging from 100 to 400 s using both pinhole collimators (0.5 and 1.0 mm diameter). *Right-hand side*: count profiles plot for the data acquired from anterior gamma images for the simulated thyroid gland (400 s) using 0.5 and 1.0 mm diameter pinhole collimators. The *yellow line* in both thyroid gamma images (400 s) represents the cross-section area of the acquired data for both count profiles

Hybrid gamma and optical images were acquired using the HGC with both pinhole collimators (0.5 and 1.0 mm in diameter) for thyroid imaging. The thyroid images produced through both pinhole collimators vary in terms of spatial resolution and number of acquired counts. Figure 8 shows the different gamma images acquired for the simulated thyroid gland utilising both pinhole collimators in a time series with acquisition time varying between 100 and 400 s. The differences between each collimator can be clearly observed, and the clarity of the acquired images improves following increasing acquisition period. At a 120 mm collimator-to-surface distance, the degradation of spatial resolution while utilising the 1.0 -mm-diameter pinhole collimator is clear from the images in Fig. 8 (left-hand side).

The count profiles through the middle of the simulated thyroid gland images provided by both pinhole collimators were obtained. Figure 8 (right-hand side) shows the difference in the spatial profiles when using the two collimators. Employing the thyroid phantom in quantitative assessment protocols would optimise the ability of the device’s operator to choose the suitable configuration of a gamma imaging system to satisfy the existing medical needs; furthermore, the phantom will be a helpful tool to determine appropriate settings for the patient and the imaging system for clinical imaging studies.

**Fig. 9:**
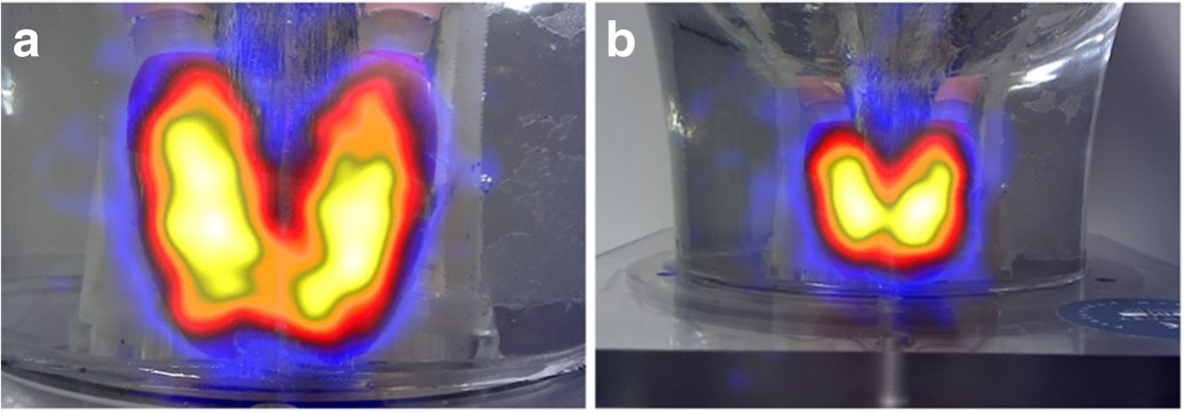
Hybrid HGC images of the simulated thyroid gland at a distance of 100 mm (**a**) and 200 mm (**b**) from the phantom surface

The HGC can produce optical-gamma fused images at different distances from the targeted tissues. Figure 9a, b represents the anterior hybrid images for the thyroid phantom from two different imaging distances (100 and 200 mm) from the camera. The acquisition time for the acquired thyroid gamma images was 400 s using the 0.5-mm-diameter pinhole collimator.

#### Simulated sentinel lymph node (SLNs) images

Imaging targeted areas intraoperatively during SLN mapping procedures requires a swift imaging process that is able to detect any potential lymph node within the field of view. To achieve this target, SFOV gamma imaging systems for intraoperative imaging should be able to provide gamma images for the targeted SLN at a short acquisition time. The anthropomorphic head and neck phantom was employed to simulate a situation where there are four SLNs distributed at two different vertical levels and depths. Using both pinhole collimators and with the HGC position at a distance of 80 mm from the surface of the phantom, several gamma images were acquired from the lateral view at different acquisition times ranging from 10 to 300 s.

**Fig. 10:**
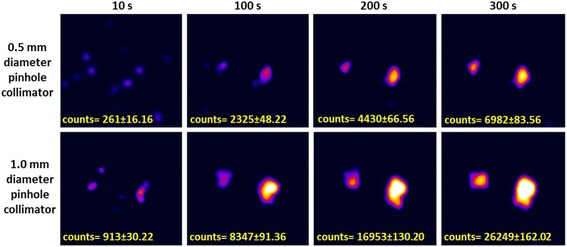
Planar HGC gamma images for the two simulated, superficial SLNs (0.1 and 0.2 MBq) at an 80 mm collimator-to-surface distance utilising both pinhole collimators (0.5 and 1.0 mm diameter); the acquisition time varied between 10 and 300 s

**Fig. 11:**
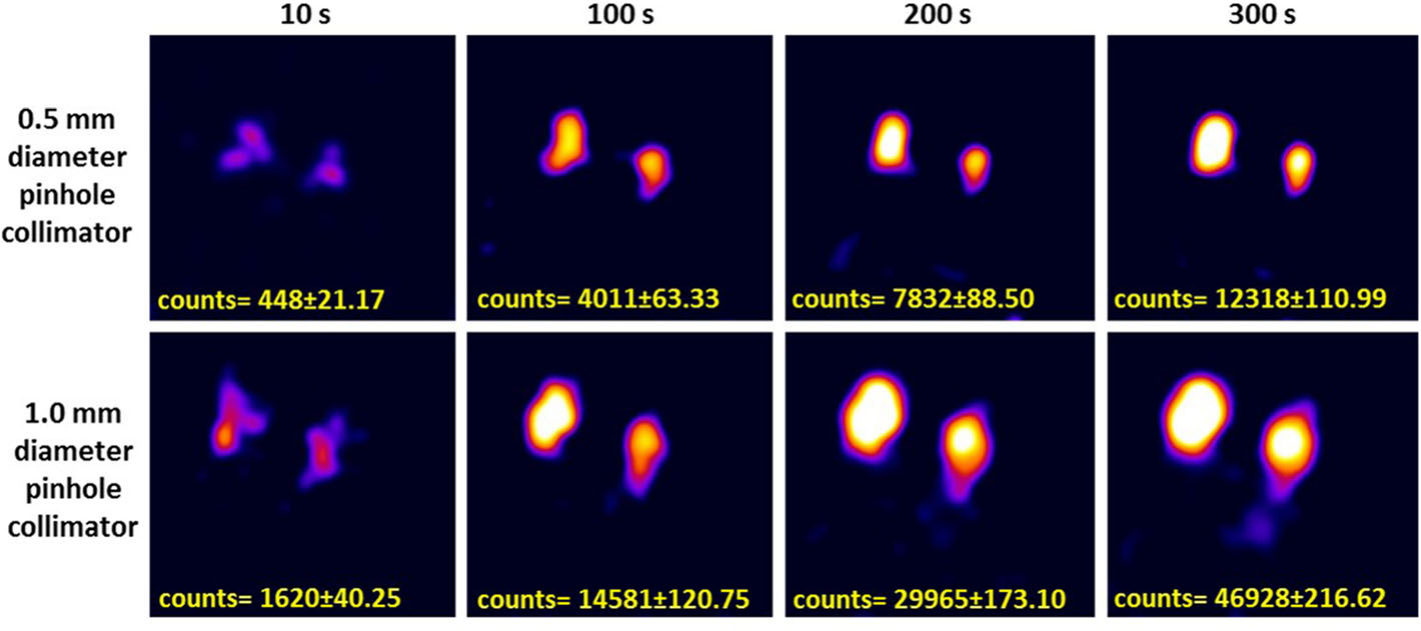
Planar HGC gamma images for the two simulated, deep SLNs (0.5 and 1.0 MBq) at an 80 mm collimator-to-surface distance; both pinhole collimators (0.5 and 1.0 mm diameter) were used, and the acquisition time varied between 10 and 300 s

**Fig. 12:**
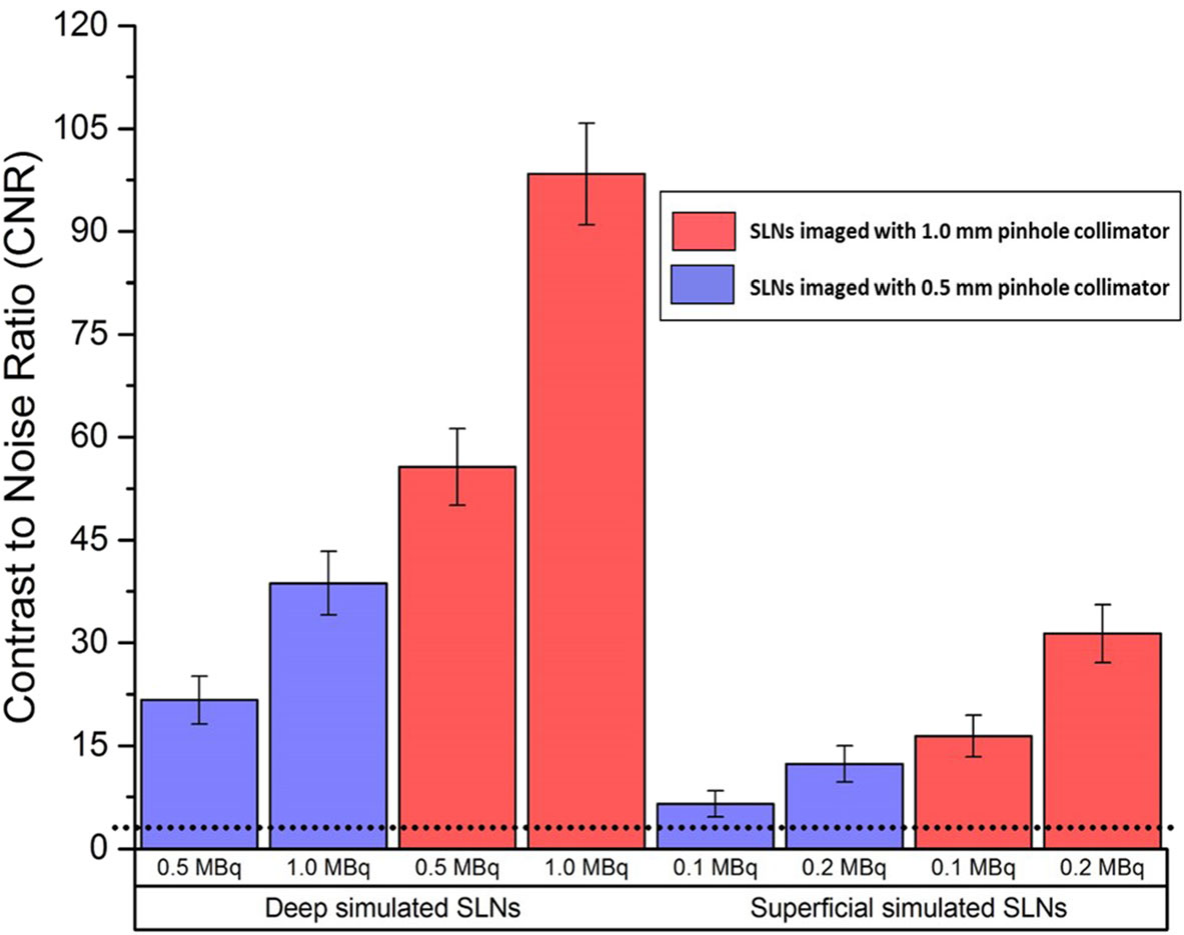
Bar chart showing the recorded contrast to noise ratio (CNR) values of different radioactivity concentrations for the simulated superficial and deep SLNs (i.e. 15 and 30 mm depth) for a 100 s acquisition time. *Error bars* represent the standard deviation of the CNR calculation for each node. The *dotted line* at CNR = 3 represents a threshold value of the Rose criterion of detectability

Figure 10 shows gamma images of the two simulated superficial SLNs at the parotid gland level containing two different radioactivity concentrations (i.e. 0.1 and 0.2 MBq). The HGC is able to clearly detect both simulated SLNs using the 1.0-mm-diameter pinhole collimator at a short acquisition time (i.e. 100 s). In contrast, the ability to detect these simulated SLNs degrades when the HGC is fitted with the 0.5-mm-diameter pinhole collimator. However, using longer acquisition times helps to provide a detailed image with an acceptable level of detectability (Fig. 10–upper row). These images show the advantage of using the 1.0-mm-diameter pinhole collimator for the imaging of low activity accumulation targeted areas at short acquisition times, as the main purpose of utilising SFOV gamma imaging systems intraoperatively is to localise the site of SLNs. This demonstrates that the HGC is capable of detecting a small amount of accumulated activity (0.1 MBq) in a reasonable acquisition time (i.e. <100 s). For higher activity accumulation targets, using the 0.5-mm-pinhole collimator (smaller diameter) will provide better spatial resolution with a good level of detectability, i.e. beyond the threshold value based on the Rose criterion of detectability (Figs. 11 and 12).

As a further measure, CNR calculations were performed for the detected SLNs at an 80 mm distance between both the pinhole collimators and surface of the head and neck phantom for a 100 s acquisition time (Fig. 12). The recorded CNR values for the simulated SLNs having low activity accumulations are higher when the HGC was fitted with the 1.0-mm-diameter pinhole collimator. For instance, the recorded CNR values for the superficially simulated SLN (15 mm depth) containing 0.1 MBq of ^99m^Tc using 0.5- and 1.0-mm-diameter pinhole collimators are 6.48 and 16.42, respectively (~87% difference).

## Discussion

Phantoms are commonly used to assess nuclear imaging devices. These range from relatively crude arrangements consisting of tanks with chambers and spheres through to software generated phantoms for the evaluation of image processing. The use of an anthropomorphic phantom has the advantage of providing accurate anatomical and functional detail which may be used for both the assessment of equipment and user training.

The current development in computer-aided design (CAD) and 3D printing machines has facilitated the process of constructing inexpensive complex 3D medical phantoms which can be patient specific. The combination of computer aided modelling, advanced medical imaging technologies (i.e. computed tomography scanner, ultrasound devices etc.) and rapid prototyping provided by 3D printers has improved the ability to fabricate objects comparable to human parts in micro and macro architecture scales. Future developments could include the insertion of 3D printed lesions or tumours using information obtained from CT or magnetic resonance (MR) medical images.

The images presented show the flexibility of the head and neck phantom to simulate many clinical scenarios; i.e. lymph nodes can be simulated at any selected position following lymphatic drainage pathways in the head and neck regions.

This work was mainly aimed at assessing the capabilities of SFOV cameras; however, the phantom could equally be used with LFOV SPECT-CT cameras. Currently, there are many SFOV gamma imaging systems employed in intraoperative imaging [11, 39, 40]. However, anthropomorphic phantoms have still not been standardised or utilised to quantify the capability of these different systems to detect targeted tissues during intraoperative procedures such as SLN mapping. Furthermore, the imaging of small organs is an area in which portable SFOV gamma cameras can provide further flexible techniques comparable to the conventional nuclear scanning techniques in cases like thyroid scans and lacrimal drainage procedures [13, 35, 36].

This study has shown the suitability of the HGC for small organ imaging such as thyroid imaging and lymph node detection in head and neck. The ability to interchange collimators helps the user prepare the HGC according to the purpose the study; for example, in cases where good spatial resolution is a requirement, such as thyroid imaging, using a 0.5-mm-diameter pinhole collimator would be suitable for a reasonable acquisition time. Nevertheless, in critical or time-dependent cases where sensitivity is of particular importance, like SLN mapping, the 1.0-mm-diameter pinhole collimator would be the proper choice. Therefore, the ability of the HGC to utilise both pinhole collimators enhances its practicality and improve its ability to meet the needs of SFOV gamma imaging systems.

The standardisation of a test protocol for SFOV portable gamma systems will provide an opportunity to collect data across various medical centres and research groups. Moreover, it will contribute towards a technical baseline for researchers and clinical practitioners to consider when assessing their SFOV gamma imaging systems.

## Conclusions

In this study, a novel anthropomorphic head and neck phantom was designed and fabricated. The internal parts and the outer shell of the phantom provided life-size adult head and neck, thyroid gland, trachea and cervical spine. In addition, different SLNs at various depths and locations, having any desired activity concentration, could be simulated. The anatomical structure of the anthropomorphic head and neck phantom was demonstrated using a SPECT-CT imaging.

This phantom was employed to evaluate the capability of a novel SFOV camera—the HGC—in simulated scenarios such as SLN mapping of the head and neck region and to show the possibility of using these gamma systems in small organ imaging such as thyroid imaging procedures.

The performance of a novel HGC was investigated using the head and neck phantom. Both pinhole collimators (0.5 and 1.0 mm diameter) were utilised, and a comparison between their performances during various gamma imaging scenarios was carried out. The results showed the ability of the HGC to image small organs, such as the thyroid gland, and to detect lymph nodes in SLN mapping procedures. The phantom provides a valuable tool for assessing camera imaging abilities prior to use in the surgical settings.

## Abbreviations

3D: Three-dimension
ABS: Acrylonitrile butadiene styrene
CAB: Cellulous acetate butyrate
CAD: Computer aid design
CNR: Contrast to noise ratio
CT: Computed tomography
EMCCD: Electron multiplying charge coupled device
HGC: Hybrid gamma camera
LFOV: Large field of view
MR: Magnetic resonance
OSEM: Ordered-subsets expectation maximisation
PMMA: Polymethyl methacrylate
ROI: Region of interest
SFOV: Small field of view
SLN: Sentinel lymph nodes
SPECT/CT: Single-photon emission computed tomography/computed tomography
